# Suture reinforcement using a modified cyanoacrylate glue to prevent anastomotic leak in colorectal surgery: a prospective multicentre randomized trial: The** Re**ctal** A**nastomotic sea**L** (ReAL) trial

**DOI:** 10.1007/s10151-024-02967-7

**Published:** 2024-08-05

**Authors:** G. Tomasicchio, G. Martines, N. Tartaglia, M. Buonfantino, E. Restini, B. Carlucci, C. Giove, A. Dezi, C. Ranieri, G. Logrieco, L. Vincenti, A. Ambrosi, D. F. Altomare, M. De Fazio, A. Picciariello

**Affiliations:** 1 Department of Precision and Regenerative Medicine and Jonic Area (DiMePRe-J), General Surgery Unit “M. Rubino”, University of Bari Aldo Moro, Bari, Italy; 2 Azienda Ospedaliero Universitaria Policlinico, University of Bari, Piazza G Cesare, 11, 70124 Bari, Italy; 3 Department of Medical and Surgical Sciences, DSMC, University of Foggia, Foggia, Italy; 4 General Surgery Unit, Hospital “San Paolo”, Bari, Italy; 5 General Surgery Unit, Hospital “L. Bonomo”, Andria, Italy; 6 General Regional Hospital “F. Miulli”, Acquaviva delle Fonti, Bari, Italy; 7 General Surgery Unit, IRCCS “Saverio De Bellis”, Castellana Grotte, Italy; 8 Department of Experimental Medicine, University of Salento, Lecce, Italy

**Keywords:** Colorectal surgery, Suture reinforcement, Cyanoacrylate, Anastomotic leakage

## Abstract

**Background:**

Anastomotic leakage (AL) is the most frequent life-threating complication following colorectal surgery. Several attempts have been made to prevent AL. This prospective, randomized, multicentre trial aimed to evaluate the safety and efficacy of nebulised modified cyanoacrylate in preventing AL after rectal surgery.

**Methods:**

Patients submitted to colorectal surgery for carcinoma of the high-medium rectum across five high-volume centres between June 2021 and January 2023 entered the study and were randomized into group A (anastomotic reinforcement with cyanoacrylate) and group B (no reinforcement) and followed up for 30 days. Anastomotic reinforcement was performed via nebulisation of 1 mL of a modified cyanoacrylate glue. Preoperative features and intraoperative and postoperative results were recorded and compared. The study was registered at ClinicalTrials.gov (ID number NCT03941938).

**Results:**

Out of 152 patients, 133 (control group, *n* = 72; cyanoacrylate group, *n* = 61) completed the follow-up. ALs were detected in nine patients (12.5%) in the control group (four grade B and five grade C) and in four patients (6.6%), in the cyanoacrylate group (three grade B and one grade C); however, despite this trend, the differences were not statistically significant (*p* = 0.36). However, Clavien–Dindo complications grade > 2 were significantly higher in the control group (12.5% vs. 3.3%, *p* = 0.04). No adverse effects related to the glue application were reported.

**Conclusion:**

The role of modified cyanoacrylate application in AL prevention remains unclear. However its use to seal colorectal anastomoses is safe and could help to reduce severe postoperative complications.

**Supplementary Information:**

The online version contains supplementary material available at 10.1007/s10151-024-02967-7.

## Introduction

Managing anastomotic leakage (AL) remains challenging for colorectal surgeons. The incidence of AL is estimated to be 2–4% following colon cancer resection [1] and 10–20% after rectal anastomosis [2]. AL considerably affects clinical outcomes with an increased risk of permanent stoma, urinary, defaecatory, and sexual dysfunction, thereby increasing health care expenditure [3, 4]. Moreover, AL increases the risk of local recurrence and decreases overall survival by delaying the initiation of adjuvant cancer treatments [5, 6].

The aetiology of AL is complex and multifactorial. Many preoperative risk factors [7], including patient-related factors (such as sex, malnutrition, obesity, American Society of Anesthesiologists (ASA) score, smoking history, cardiovascular disease, nonsteroidal anti-inflammatory drugs usage, neoadjuvant chemoradiotherapy, age, altered gut microbiota), cancer-related factors (stage, distance from the anal verge), and surgeon-related factors [8, 9], such as the surgeon’s expertise and experience, type of anastomosis, and use of faecal diversion by temporary stoma, are implicated [10, 11].

Therefore, adopting preventive measures is highly recommended. Although patient- and cancer-related risk factors cannot be modified, several intraoperative procedures have been implemented to prevent this serious complication, such as indocyanine green injection to assess adequate perfusion of the distal colon, reinforcement of the anastomosis by additional manual stitches and/or collagen patches or sealants, and suture protection with an omental flap [12–16].

Among the possible options to reinforce and protect anastomosis, cyanoacrylate glue application has significantly reduced AL in colon anastomosis in a porcine model [17–20] and prevents suture leakage after sleeve gastrectomy [21]. Furthermore, cyanoacrylate is widely used in endoscopy for the emergency management of patients with upper gastrointestinal bleeding because of its adhesive and haemostatic seal when nebulised and sprayed on injured tissues [22]. Furthermore, the sealing effect creates an antiseptic barrier against bacteria [23]. The application of cyanoacrylate glue in vascular surgery [24], urology [25], and bariatric surgery [21] has been previously described.

Considering its mechanical, physical, and biological properties and safety, cyanoacrylate glue can facilitate the healing of colorectal anastomoses and reduce the AL rate, as demonstrated in experimental and uncontrolled clinical studies [26].

The distance of the anastomosis from the anal verge is a well-recognised risk factor for AL; therefore, low/ultralow anastomosis is generally protected by a covering ileostomy, whereas anastomoses performed on the intraperitoneal tracts of the colon and rectum are considered to have a low AL risk. Therefore, using a covering stoma to protect the surgical anastomosis involving the upper part of the rectum remains ambiguous, and is often left to the discretion of the surgeon.

This prospective, randomized, multicentre trial aimed to evaluate the role of a nebulised modified cyanoacrylate in preventing AL in colorectal surgery.

## Patients and methods

A prospective, multicentre, parallel-arm, randomized, controlled superiority trial was conducted between June 2021 and January 2023 across five high-volume tertiary referral centres for colorectal surgery. A power analysis was conducted to determine the number of patients enrolled in each study arm. The study protocol was approved by all local ethics committees in accordance with the Declaration of Helsinki and registered at ClinicalTrials.gov (ID number NCT03941938).

This study included consecutive patients diagnosed with histologically confirmed primary adenocarcinoma of the high-medium rectum without internal and/or external sphincter muscle involvement, with a distal margin of the tumour at least 8 cm from the anal verge, stage T2–T4 on magnetic resonance imaging (MRI) before neoadjuvant chemoradiation. Patients with squamous cell carcinoma, stage T1 or T4 adenocarcinoma with pelvic side wall involvement or requiring sacrectomy or prostatectomy (partial or total), unresectable primary rectal cancer or inability to complete R0 resection, rectal cancer less than 8 cm from the anal verge requiring coloanal or ultra-low rectal anastomosis, recurrent rectal cancer, previous pelvic malignancy, inability to return for postoperative follow-up, and inability to sign informed consent were excluded. Preoperative colonoscopy of the entire colon, computed tomography (CT) scan staging, and preoperative tumour and nodal staging using MRI and/or endorectal ultrasonography were performed for all participants. Surgery was performed within 8–12 weeks (56–84 days) after the completion of neoadjuvant therapy, when indicated. All patients were fully informed about the study, and informed consent for inclusion in the study was obtained. The participants were informed of their right to withdraw from the study or refuse initial enrolment at any point.

### Randomization

Computer-generated randomization was used to create an allocation sequence to assign patients to different study arms. Randomization was centrally controlled by an operator who was not involved in the study. Patients were randomized only after the completion of neoadjuvant chemotherapy (when indicated) and before surgery. Patients randomized to the cyanoacrylate glue arm received anastomotic reinforcement with nebulised modified cyanoacrylate glue, whereas no anastomotic reinforcement was used in the control group. The modified cyanoacrylate used to reinforce the anastomosis is Glubran®2 (GEM S.r.l., Viareggio, Italy), a synthetic surgical biodegradable cyanoacrylate-based glue (NBCA + MS), modified by the addition of a monomer metacryloxysulfolane (MS) into* n*-butyl-cyanoacrylate (NBCA). As part of the educational intervention, all surgeons underwent training using a video demonstrating the surgical technique for the application of nebulised NBCA + MS.

### Operative protocol

Patient preparation for surgery followed the general rules for Good Clinical Practice. Mechanical bowel preparation using an orally administered polyethylene glycol solution was performed in all patients, and antibiotic and antithrombotic prophylaxis were started immediately before surgery. The patients were placed in the lithotomy position for the abdominal and perineal procedures. Abdominal procedures (open or laparoscopic) were performed according to the oncological guidelines, including total mesorectal excision with adequate lymph node retrieval and at least 1 cm of distal clearance with an end-to-end or latero-terminal Knight–Griffen anastomosis. A pneumohydraulic test was performed to check for AL, and additional sutures were applied in positive tests. In patients randomized for the anastomotic reinforcement, the modified cyanoacrylate-based glue (NBCA + MS) was then nebulised all around the anastomosis for 2–3 s using the related special catheters for open or laparoscopic surgery.

Following the enhanced recovery after surgery protocol [27], the nasogastric tube was removed in the operating room after surgery completion, and the urinary catheter was removed within 72 h for rectal resection. Early mobilisation was strongly encouraged in all patients on the first postoperative day. Patients were allowed to drink clear liquids in the postoperative period once awake and were able to drink safely.

### Postoperative management

Postoperative care was provided in accordance with the current standards directed by the operative surgeon. Narcotics or analgesics for pain were initially administered through the parenteral route (intramuscular, intravenous, or epidural), followed by oral administration when the patient resumed oral intake. Oral intake was allowed according to the patients’ tolerance. The patients returned to the ambulatory clinic for postoperative follow-up after 30 days. During follow-up, the postoperative data were recorded, and proctography was performed by transanal injection of 100 mL of hydro-soluble contrast medium (Gastrografin, sodium amidotrizoate and meglumine amidotrizoate, Bayer S.p.A.) to check the integrity of the anastomosis. AL was graded according to the classification system developed by Rahbari et al. Patients who did not attend the follow-up were contacted by a research nurse by phone or email, where available, to ascertain whether the patient had experienced any complications and/or adverse events treated in other hospitals. Patients unavailable for postoperative evaluation were considered lost to follow-up.

### Outcomes measures

Demographic data (age, sex, weight, BMI (kg/m^2^), ASA classification, smoking status, and comorbidities) and intraoperative data (type of operative approach, operative time, type of anastomosis, and sutures) were recorded. Postoperative complications were classified in accordance with the Clavien–Dindo classification [28] and recorded along with the length of in-hospital postoperative days, 30-day readmission rate, and death. AL was defined as a defect in the intestinal wall integrity at the colorectal anastomotic site, leading to communication between the intra- and extraluminal compartments [29], with the presence of faecal discharge from the pelvic drainage at any time after surgery, rectovaginal fistula (defined as faecal or mucus discharge from the vagina), and pelvic sepsis (defined by the collection of pus/faecal material in the pelvis documented by a CT scan). ALs were classified according to the Rahbari et al. classification: grade A, AL requiring no active therapeutic intervention; grade B, AL requiring active therapeutic intervention but manageable without relaparotomy; and grade C, AL requiring re-laparotomy [29].

### Statistical analysis

#### Sample size determination

The expected mean percentage of AL after rectal cancer is 16%, and a reduction to 10% was considered clinically significant. The sample size calculation determined that 67 patients per arm was sufficient to reject the null hypothesis with a power of 0.8 and a type I error probability of 0.05, with a confidence level of 95% (sample size calculated by R Studio Version 1.1.419, ©2009–2018 RStudio, Inc.). To account for a predicted 5% estimated loss to follow-up, the sample size was calculated as 138 (134 + 4).

#### Statistical methods

All statistical analyses were performed in an intention-to-treat manner. No interim analyses were planned. A *P* value of 0.05 indicated statistical significance. Student’s *t* test was used to provide an unadjusted estimate of the difference between treatment arms. Analysis of covariance, a more robust method that allows the control of key differences in baseline characteristics, was used to enable adjusted comparisons.

Similar to the primary outcome, continuous secondary outcomes (such as safety and efficacy) were modelled using *t* tests and analysis of covariance. The relationship between AL and other possible adverse factors was evaluated using univariate and multivariate regression analyses. When the complete case analysis excluded more than 5% of patients owing to missing data, exploratory analyses were performed to investigate the effect of missing data. To explore the mechanism of missing data and the validity of a complete case analysis for each endpoint, patient characteristics were compared between those with and without missing data, and multilevel logistic regression models were used to identify any associations between prognostic variables and determine if data were missing at random. The prognostic effect of each baseline parameter on AL outcomes (present/absent) was analysed using logistic regression. All parameters were analysed individually using logistic regression, and parameters with a probability level less than 0.1 were selected for analysis using stepwise multivariable regression. A polychotomous stepwise logistic regression model was used to correlate prognostic factors with Clavien–Dindo classification grades. Multilevel logistic regression was used to estimate the odds ratios (ORs) for conversion to laparotomy, intraoperative complications, and postoperative complications between the treatment groups.

## Results

**Fig. 1:**
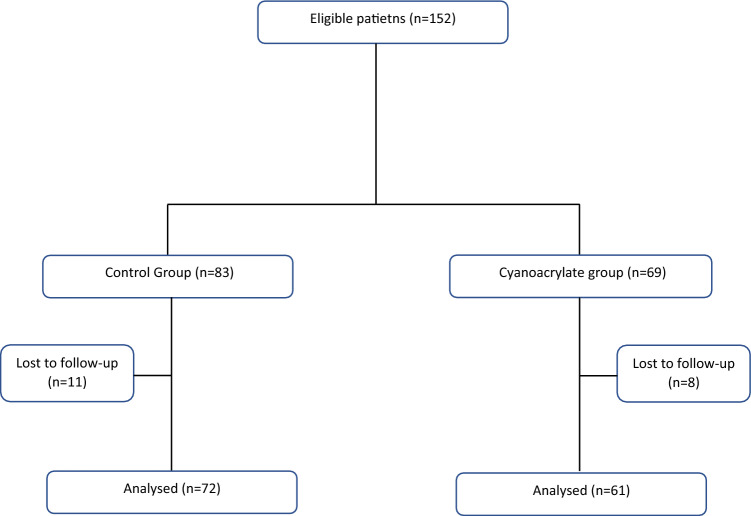
CONSORT diagram of patients in the control and cyanoacrylate groups

This study included 152 patients with primary adenocarcinoma of the high-medium rectum who were randomized into two arms. Following the completion of 30-day postoperative follow-up by 133 patients (Fig. 1), 72 and 61 were randomized into the control (median age 68, interquartile range [IQR] 58–76.5; 34.7% women) and the anastomotic reinforcement groups (median age 72, IQR 61–77; 47.5% women), respectively. No statistically significant differences in baseline characteristics were observed between the two groups (Table 1). However, the operative approach significantly differed between the two groups; 53 patients (86.9%) in the anastomotic reinforcement group underwent a laparoscopic approach compared to 51 patients (70.8%) in the control group (*p* = 0.0255). Moreover, the operative time was significantly longer in the control group than in the anastomotic reinforcement group (180 min, IQR 150–240 vs. 160, IQR 135–180, respectively; *p* = 0.0129). No conversion to laparotomy was reported in patients who had laparoscopic intervention. The end-to-end anastomosis was the most frequent type of anastomosis performed in both groups (65.3% vs. 78.7%), without statistical difference (*p* = 0.088). Only two patients (1.5%) in the control group had a hand-sewn anastomosis. Only three patients received neoadjuvant radiochemotherapy (one in the Glubran group and two in the controls), and none of them had AL.

**Table 1:** Baseline characteristics of patients in the control and cyanoacrylate groups

	Control *n* = 72	Cyanoacrylate *n* = 61	*p* value
Age (years)	68 (58–76.5)	72 (61–77)	0.30
Sex			
Female	25 (34.7%)	29 (47.5%)	0.13
Male	47 (65.3%)	32 (52.5%)	
BMI (kg/m^2^)	27 (24.4–30)	26 (23.5–28.7)	0.07
Comorbidities			
Smoker	11 (15.3%)	17 (27.9%)	0.07
Cardiopathy	15 (20.8%)	14 (23%)	0.76
Hypertension	42 (58.3%)	13 (18.1%)	0.29
Diabetes	30 (49.2)	12 (19.7%)	0.81
ASA			
I	2 (2.8%)	3 (4.9%)	0.83
II	46 (63.9%)	41 (67.2%)	
III	23 (31.9%)	16 (26.2%)	
IV	1 (1.4%)	1 (1.6%)	

Linear Contour or Echelon staplers (Ethicon, J&J) were used to close the rectal stump in case of open or laparoscopic approach, respectively, whereas 28 or 31 mm circular staplers from Covidien or Ethicon Endosurgery were used according to the size of the sigmoid lumen and the device availability in the different hospitals.

The air leak test was positive in one patient (1.4%) in the control group and five patients (8.2%) in the anastomotic reinforcement group (*p* = 0.059), and all patients had hand suture reinforcement.

**Fig. 2:**
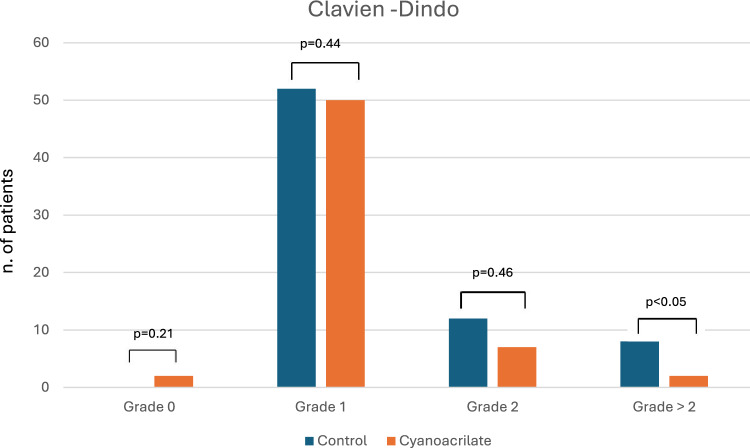
Clavien–Dindo grade and comparison of control and cyanoacrylate groups

A protocol deviation with a temporary protective ileostomy was performed in five (6.9%) and six (9.8%) patients in the control and anastomotic reinforcement groups, respectively, with no statistically significant difference (*p* = 0.54). No significant difference was observed in the duration of in-hospital stays between the two groups (7.5 days, IQR 6–10 vs. 7 days, IQR 6–9; *p* = 0.23). Clavien–Dindo complications (grade > 2) were significantly higher in the control group (9 [12.5%] vs. 2 [3.3%]; *p* = 0.04) (Fig. 2). Four patients in the control group had Clavien–Dindo grade 3 complications; one had grade 3A, and three had grade 3B, while only one patient in the cyanoacrylate group had grade 3B, which required intervention under general anaesthesia. Fatal complications requiring intensive care unit management were reported in two patients in the control group, one with single-organ failure (grade 4A) and one with multi-organ failure (grade 4B). None of the patients in the anastomotic reinforcement group experienced grade 4 complications. Mortality due to postoperative complications was reported in one patient in both groups (grade 5).

ALs were observed in nine patients (12.5%): four grade B and five grade C in the control group vs. four patients (6.6%), three grade B and one grade C in the anastomotic reinforcement group; however, this difference was not statistically significant (*p* = 0.36). No adverse effects due to the application of nebulised NBCA + MS around the colorectal anastomosis were reported. No significant differences in 30-day readmission and postoperative death rates were observed between the groups (Table 2).

**Table 2:** Operative and postoperative data of patients in the control and cyanoacrylate groups

	Control *n* = 72	Cyanoacrylate *n* = 61	*p *value
Operative approach			
Open	21 (29.2%)	8 (13.1%)	0.025
Laparoscopy	51 (70.8%)	53 (86.9%)	
Type of anastomosis			
LT	25 (34.7%)	13 (21.3%)	0.088
TT	47 (65.3%)	48 (78.7%)	
Temporary ileostomy			
Yes	5 (6.9%)	6 (9.8%)	0.54
No	67 (93.1%)	55 (90.2%)	
Type of suture			
Hand	2 (2.8%)	0	0.189
Stapler	70 (97.2%)	61 (100%)	
Air leak test			
Positive	1 (1.4%)	5 (8.2%)	0.06
Negative	71 (98.6%)	56 (91.8%)	
Postoperative stay (days)	7.5 (6–10)	7 (6–9)	0.23
Anastomotic leak grade^a^			
B	4 (5.5%)	3 (5%)	0.22
C	5 (7%)	1 (1.6%)	
Clavien–Dindo			
0	0	2 (3.3%)	0.04
1	51 (70.9%)	50 (82%)	
2	12 (16.6%)	7 (11.5%)	
> 2	9 (12.5%)	2 (3.3%)	
Readmission at 30 days			
Yes	2 (2.8%)	1 (1.6%)	0.65
No	70 (97.2%)	60 (98.4%)	
Postoperative death			
Yes	1 (1.4%)	1 (1.6%)	0.9
No	71 (98.6%)	60 (98.4%	

^a^According to Rahbari et al.’s classification [29]

None of the parameters analysed using logistic regression and/or stepwise multivariate regression were statistically significant for AL. Among them, heart disease increased the risk of leakage by 2.89 times (OR 2.89; 95% CI 0.84–9.89), which was not statistically significant (*p* = 0.091).

Concerning the Clavien–Dindo grade > 1 complications, patients with higher ASA scores (> 2) are at significantly increased risk (three times higher) of developing complications (OR 3.35, 95% CI 1.37–8.18) than those in the control group (*p* < 0.01).

The risk of developing Clavien–Dindo grade > 1 complications was lower in women than in men (OR 0.41, 95% CI 0.15–1.08). Moreover, two patients who had undergone hand-sewn anastomosis experienced five times higher risk than those who underwent stapler procedures to develop Clavien–Dindo complications grade > 1 (OR 8.7, 95% CI 0.64–119); however, the difference for both parameters was not statistically significant.

## Discussion

Anastomotic leakage remains the most fatal postoperative complication in colorectal surgery. Several sealants and tissue adhesives have been investigated in animal and human models to limit the incidence of AL [30]. NBCA + MS, classified by the manufacturer as a class III medical device, possesses haemostatic and adhesive features [31]. Upon solidification, it forms an antiseptic barrier in surgical settings [32]. When NBCA + MS glue comes into contact with vital tissue, it creates an elastic layer with high tensile strength [31], ensuring firm adhesion conforming to the anatomy of tissue [23].

Since its introduction, cyanoacrylate has gained considerable attention as an anastomotic sealant in colorectal surgery, with controversial results [33–35]. Most studies were animal experiments that reported positive outcomes. Few clinical studies have been conducted on patients undergoing bowel anastomosis. D’Amore et al. conducted a single-centre retrospective, uncontrolled study to assess the safety of Glubran®2 for colorectal anastomosis in humans [36]. Their findings revealed AL in only one patient (0.9%), a rate which was lower than that observed in our study. However, they included patients with histological diagnoses of cancer in all possible colorectal locations, and different types of surgical procedures, and lacked a control group for comparison.

To our knowledge, this study is the first randomized controlled multicentre study investigating the efficacy of nebulised NBCA + MS in preventing colorectal AL. Our findings indicate that only 6.6% of the patients treated with this glue developed AL, compared to 12.5% in the control group; however, the difference was not statistically significant, suggesting the possibility of a type II error in the sample size calculation. However, the incidence of Clavien–Dindo complications (grade > 2) was significantly lower in the cyanoacrylate group (*p* = 0.04). Consistent with previous studies [26, 36], no adverse reactions to the modified cyanoacrylate were recorded in our patient cohort, highlighting its safety as an anastomotic sealant in clinical settings.

The effectiveness of other biological sealants in preventing AL has been evaluated in clinical settings, and most studies reported a decreased AL rate in their intervention group, suggesting a beneficial effect on colorectal anastomosis healing [26]. However, only Lago Oliver et al. [37], in a randomized, single-blind, parallel study, found a statistically significant difference (18.8% vs. 52.5%, *p* = 0.039) using fibrin-based biological adhesive (Tissucol Duo®, Baxter AG, Vienna, Austria) in patients who underwent rectal resection. However, the high incidence of AL reported in their small sample size compared to that in the literature may have influenced the outcome of their statistical analysis. Furthermore, Valsamidis et al. [26] in their systematic review emphasised that variations in defining, identifying, and detecting AL between studies introduce heterogeneity that undermines the reliability of the results. To overcome this limitation, our study adopted the Rahbari et al. classification for AL and used a widely accepted classification of postoperative complications such as the Clavien–Dindo classification [26]. Shamiyeh et al. [38] reported a complication rate of 5.7% classified as Clavien–Dindo III/IV using Obsidian ASG® autologous platelet-rich fibrin matrix. However, that study did not include controls.

Our study had certain limitations. First, the low statistical power (0.80) and the risk of type II error for the relatively low number of patients recruited, although this is counterbalanced by stringent eligibility criteria. Moreover, protocol deviations occurred in 11 patients who underwent a temporary protective ileostomy at the discretion of the surgeon. Furthermore, the short-term follow-up cannot account for any long-term oncological outcomes caused by sealants.

## Conclusions

The application of the modified cyanoacrylate to seal colorectal anastomoses could be considered safe and could contribute to reducing severe postoperative complications; however, its role in preventing AL remains unclear.

## Supplementary Information

Below is the link to the electronic supplementary material.

Supplementary file1 (DOC 220 KB)

## Data Availability

All data generated or analysed during this study are included in this article. Further enquiries can be directed to the corresponding author.
